# Mental Health of Adolescents Exposed to the War in Ukraine

**DOI:** 10.1192/j.eurpsy.2025.140

**Published:** 2025-08-26

**Authors:** N. Skokauskas

**Affiliations:** NTNU, Trondheim, Norway

## Abstract

**Abstract:**

This presentation will provide an overview of our original studies focusing on the mental health impact of the ongoing war in Ukraine on adolescents. Our studies address critical areas such as posttraumatic stress disorder (PTSD), anxiety, depression, suicidality, and the prevalence of psychiatric conditions, alongside resilience factors among war-affected youth. By synthesizing findings from the studies , the presentation will hilight the substantial psychological burden of the conflict on Ukrainian adolescents and emphasizes the pressing need for targeted mental health interventions. These studies contribute essential insights into the long-term implications of trauma, resilience, and mental health outcomes in conflict-affected populations, informing both research and clinical practice.

**Disclosure of Interest:**

None Declared

